# Bypass during Liver Transplantation: Anachronism or Revival? Liver Transplantation Using a Combined Venovenous/Portal Venous Bypass—Experiences with 163 Liver Transplants in a Newly Established Liver Transplantation Program

**DOI:** 10.1155/2015/967951

**Published:** 2015-03-02

**Authors:** Anne Mossdorf, Florian Ulmer, Karsten Junge, Christoph Heidenhain, Marc Hein, Ilknur Temizel, Ulf Peter Neumann, Wenzel Schöning, Maximilian Schmeding

**Affiliations:** ^1^Department of General, Visceral and Transplantation Surgery, University Hospital Aachen, Pauwelsstrasse 30, 52074 Aachen, Germany; ^2^Department of Anaesthesiology, University Hospital Aachen, Pauwelsstrasse 30, 52074 Aachen, Germany; ^3^Department of Internal Medicine III, University Hospital Aachen, Pauwelsstrasse 30, 52074 Aachen, Germany

## Abstract

*Introduction*. The venovenous/portal venous (VVP) bypass technique has generally become obsolete in liver transplantation (LT) today. We evaluated our experience with 163 consecutive LTs that used a VVP bypass. *Patients and Methods*. The liver transplant program was started in our center in 2010. LTs were performed using an extracorporal bypass device. *Results*. Mean operative time was 269 minutes and warm ischemic time 43 minutes. The median number of transfusion of packed cells and plasma was 7 and 14. There was no intraoperative death, and the 30-day mortality was 3%. Severe bypass-induced complications did not occur. *Discussion*. The introduction of a new LT program requires maximum safety measures for all of the parties involved. Both surgical and anaesthesiological management (reperfusion) can be controlled very reliably using a VVP bypass device. Particularly when using marginal grafts, this approach helps to minimise both surgical and anaesthesiological complications in terms of less volume overload, less use of vasopressive drugs, less myocardial injury, and better peripheral blood circulation. *Conclusion*. Based on our experiences while establishing a new liver transplantation program, we advocate the reappraisal of the extracorporeal VVP bypass.

## 1. Introduction

Liver transplantation (LT) has become a routine procedure for acute liver failure and chronic liver diseases of variable origins today. The explantation of the entire organ, including the retrohepatic vena cava and caval replacement (CR), represented the standard operative procedure in the early years of LT. For this procedure, a venovenous bypass shunting the blood flow from the femoral to the axillary vein was often used to relieve venous congestion in the lower parts of the body. An additional cannula was often employed to shunt the portal venous blood into the extracorporeal circulation system. By the introduction of the piggyback (PB) technique, the complete clamping of the vena cava could be avoided, making the caval component of the extracorporeal bypass unnecessary. Because the caval anastomosis time was shortened by this technique, it was possible to reduce the period of time for the complete portal venous clamping, thereby minimising the impact of mesenteric congestion. Furthermore, by avoiding extracorporeal circulation, the operative time could be reduced, as cannulation of both the femoral/saphenic and axillary veins became obsolete. Bypass-induced complications, such as thrombotic or embolic events, were also avoided, as was using costly materials [1]. For these reasons, the vast majority of LT centres have implemented the PB technique as their standard procedure.

At the University Hospital Aachen, the liver transplant program was initiated in 2010. When starting the program, we intended to establish maximum safety measures for all of the parties involved. For this reason, we considered the use of a venovenous extracorporeal bypass technique that included the portal venous blood flow during the anhepatic phase. Under the employment of the extracorporeal bypass device an excellent control of cardiac preload as well as the complete elimination of mesenteric congestion, with potentially reduced haemorrhaging during the anhepatic phase, may contribute to increased safety. With many transplant recipients displaying Model of End-stage Liver Disease (MELD), scores above 30 and an increasing organ shortage prompting the acceptance of more marginal organs [2], we believed that the use of an extracorporeal bypass may serve to stabilise the haemodynamic conditions in these settings.

In this analysis, we present our experiences and the results of 163 consecutive liver transplants performed in a newly established LT program using an extracorporeal venovenous bypass system incorporating a portal venous bypass during the anhepatic period.

## 2. Patients and Methods

One hundred and sixty-three consecutive patients who received LTs at the University Hospital Aachen between June 2010 and December 2013 were included in this analysis. Patients with combined kidney-liver transplantations, living-donor liver transplantation, and split liver transplantation were excluded.

### 2.1. Liver Transplantation

For all of the patients, LT was performed using an extracorporeal venovenous/portal venous bypass. The bypass was established by the surgical cannulation of the left saphenic vein and left axillary vein followed by connection to a roller pump system with an adjustable pace. After preparation of the hepatoduodenal ligament and ligation of the hepatic artery, an additional cannula was inserted into the distal portal vein contributing to the portal venous blood flow to the lower body part of the bypass using a Y-shaped tube switch. After clamping both of suprahepatic and infrahepatic vena cava (VC), the liver was removed. The transplantation was performed starting with the anastomosis of the suprahepatic VC, followed by the infrahepatic VC and the hepatic artery (HA). The portal venous branch of the bypass was then disconnected, and the bypass flow was reduced. A portal venous end-to-end anastomosis was performed before the simultaneous arterial and portal venous reperfusions. After establishing stable conditions, the femoroaxillary component of the bypass was removed before the bile duct anastomosis. Cardiac function was monitored by the central venous oxygen saturation. In case of preoperative cardiac dysfunction a transesophageal echocardiography was performed.

Initial immune suppression consisted of Tacrolimus (trough level 10 *μ*g/L), Basiliximab (20 mg both intraoperatively and on day 4), and corticosteroids (250 mg intraoperatively, 1 mg/kg on day 1, decreasing).

The data on the recipients sex, age, aetiology of the disease, and laboratory (lab) MELD score were collected.

Furthermore, parameters such as the operative time, transfusion requirement, warm ischemic time (WIT), postoperative liver enzyme values, and type of organ allocation were analysed.

The following donor data were examined: sex, age, BMI, cold ischemic time (CIT), bilirubin (mg/dL), aspartate aminotransferase (AST) (U/L), alanine aminotransferase (ALT) (U/L), sodium (mmol/L), length of ICU stay, and cause of death.

Extended donor criteria (marginal organs) were defined according to the German medical association as bilirubin > 3 mg/dL, AST or ALT > 150 U/L, age > 65, intensive care unit stay > 7 days, BMI > 30, sodium > 165 mmol/L, and steatosis hepatis > 40% [2].

The outcomes of the liver transplantations were evaluated by the length of stay in the intensive care unit (ICU), duration of hospitalisation, 30-day and 1-year patient and graft survival, primary nonfunction (PNF), and number of liver retransplantations (re-LT). Following the specifications of the Eurotransplant, PNF was defined as the retransplantation or death within 14 days after an LT. Furthermore, the incidence of acute renal failure, postoperative bleeding, and vascular and biliary complications were analysed. Acute renal failure was defined as an increase in the serum-creatinine of more than 50% combined with oliguria or anuria or need of renal replacement therapy on postoperative day (POD) 1–14. Postoperative bleeding was determined as the substitution from three or more packed cells/24 h with a consecutive surgical revision.

The patients' follow-up ended in March 2014. At that time, 107 patients had been followed up for at least one year. The median follow-up was 566 days.

### 2.2. Statistics

A statistical analysis was performed with SPSS statistical software (IBM, SPSS Statistics 20, Chicago, USA). The Kaplan-Meier method was used to estimate the observed 30-day and 1-year graft and patient survival. For the continuous variables, the results are given as the median and the range (minimum and maximum).

## 3. Results

### 3.1. Recipient Characteristics

The median age was 54 years. The male-to-female ratio was 106 : 57. The median labMELD-score was 17 (6–40). Twenty-four percent of the patients had a labMELD-score > 30. The primary reasons for the liver transplantations were HCC (24%) and alcohol-induced liver cirrhosis (27%). The demographics of our patients are shown in Table 1.

### 3.2. Donor Characteristics

The median donor age was 58 years. The male-to-female ratio was 83 : 79. Forty-five percent of the donors fulfilled at least one extended donor criterion (EDC). An age > 65 and a BMI > 30 were the most frequently met EDCs. The median cold ischemic time was 7.7 hours.  Table 2 shows the donors' characteristics.

### 3.3. Intraoperative Data

The median operation time was 270 minutes, with a median warm ischemic time of 43 minutes. The patients had an intraoperative requirement of 7 packed cells and 14 fresh frozen plasmas. Forty-seven percent of the organs were allocated by a rescue-allocation procedure. There were no intraoperative death and no bypass-associated event, such as thrombosis, embolism, vascular lesion, or increased blood loss during the cannulation or disconnection. The intraoperative data are shown in Table 3.

### 3.4. Postoperative Morbidity and Mortality

The average hospital stay was 32 days. The patients had a median stay in the ICU of 5 days.

The most frequent complication was postoperative bleeding. Eleven percent of the patients had to undergo a surgical revision. In most cases, the complication was diffuse bleeding in the patients with a poor coagulation status. Vascular complications of a thrombosis of the HA and/or the PV occurred only in three cases. Two patients had a successful thrombectomy, and in 1 patient a re-LT was necessary. Bile leaks were observed in 5 patients with consecutive surgical revisions.

We observed 2 patients who had a persistent groin seroma in the venous cannula access site that required surgical revision in both cases. No inguinal or axillary nerve or vascular lesion was detected.

Despite the use of a considerable number of marginal organs (see Table 2), we observed significant postreperfusion syndromes (PRS) in only 7 cases. According to Hilmi et al. we defined significant PRS as severe hemodynamic instability such as persistent hypotension (more than 30% of the anhepatic level), asystole, or hemodynamically significant arrhythmia [13].

**Figure 1:**
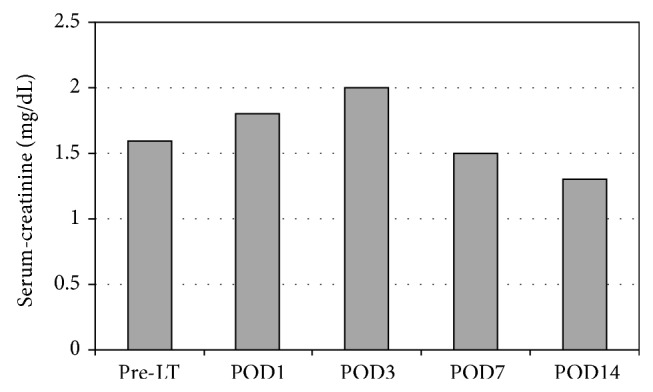
Postoperative creatinine values.

Eleven percent of the patients developed acute renal failure, 8% that required continuous venovenous hemofiltration for 2–5 days. All of the patients recovered without the need for permanent dialysis. The postoperative creatinine values are shown in Figure 1.

The number of re-LTs was 10 (4%), 5 within the first 30 days after the LT because of PNF. Two re-LTs became necessary because of ischemic type biliary lesions. One patient developed a hepatic artery and portal vein thrombosis with a consecutive re-LT. One re-LT was necessary because of the recurrence of HCV cirrhosis, and another re-LT was necessary because of metastasis to the liver of a colon carcinoma that had been transplanted with the first donor liver.

The 30-day graft survival was 93%. There was no statistically significant difference between patients with a labMELD < 30 or > 30. The 30-day patient survival was 97% (99% labMELD < 30, 90% labMELD > 30, *P* = 0.01).

**Figure 2:**
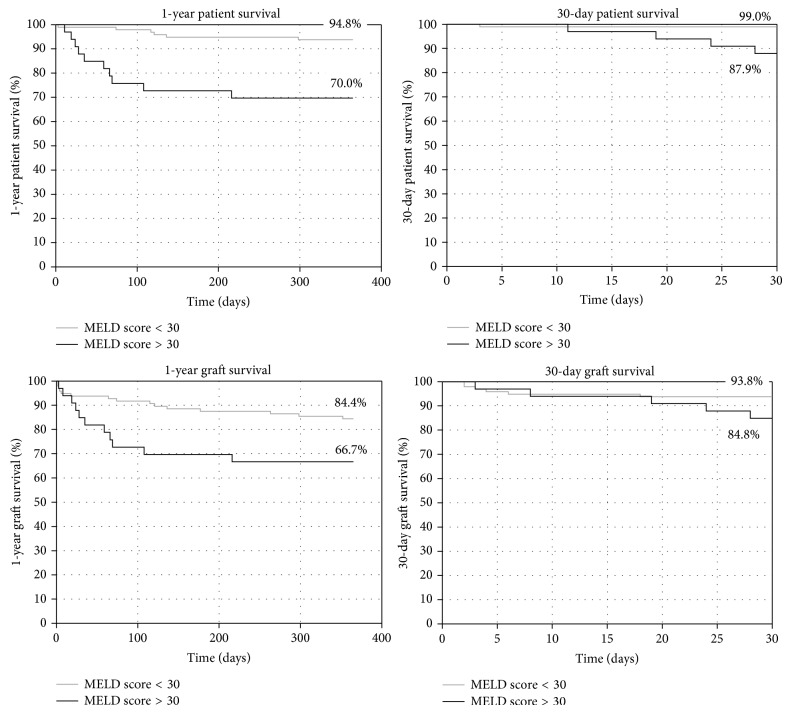
Patient and graft survival.

The one-year graft survival was 80% (85% labMELD < 30, 67% labMELD > 30, *P* = 0.04) and the one-year patient survival was 88% (94% labMELD < 30, 70% labMELD > 30, *P* = 0.001). The graft and patient survival are depicted in Figure 2.

The postoperative complications are shown in Table 4.

## 4. Discussion

The use of an extracorporeal venovenous bypass during liver transplantation has generally become obsolete in LT programs. The introduction of the PB technique for venous anastomosis with preserved caval flow has increasingly gained popularity. This technique seems to be associated with less renal function impairment, reduced blood product requirements, and shorter WIT compared with the CR strategy [5, 8–12, 14].

Numerous studies have been performed on this topic; however, most of them are retrospective in nature and single-centred. Gurusamy et al. performed a Cochrane review in 2011. Due to the heterogeneity of the available data, the authors were able to include only three studies that fulfilled the inclusion criteria. In their analysis, no significant differences concerning postoperative kidney function and the amount of blood products required could be detected. Neither the bypass-associated morbidity nor the patient or graft survival was evaluated [15].

Consequently, some vagueness remains concerning the question of whether the employment of a venovenous bypass including the portal venous axis may have advantages in terms of the spared mesenteric congestion and the consequently increased safety during transplantation and reperfusion. Most studies comparing the PB technique to CR with or without using a bypass analysed a setting in which the portomesenteric compartment was not included in the extracorporeal circulation [7, 9, 10]. This, however, may be the most important positive aspect of the bypass procedure: By avoiding mesenteric congestion during the portal venous clamping, the intensity of diffuse abdominal bleeding can be reduced; additionally, the cardiac preload may be adjusted comfortably by reducing or increasing the blood flow of the bypass. The somewhat time-consuming procedure of bypass installation may therefore be compensated for by the reduced necessity of combatting haemorrhages. In our series, we did not detect an increased operative time compared with the data in the current literature, despite the additional 20–30 minutes required for the groin and axilla preparation, cannulation, bypass connection and disconnection, and closure of the additional incisions.

The reduced transfusion requirement is often reported as a benefit of the PB technique compared with a CR. Our data demonstrate that the intensity of the blood product transfusion in our series was well within the international standards [3, 4].  Table 5 shows a comparison of data described in the literature and our data.

Several studies comparing the PB strategy to CR report improved postoperative renal function as one of the assets of the PB technique. However, these data are partly distorted by the fact that the PB technique was compared to CRs both with and without bypasses. As the total caval clamping induces venous renal congestion, it is not surprising that the PB technique with a preserved caval flow shows improved postoperative GFR. Cabezuelo et al. describe renal failure in up to 50% of LT with CR and bypass [9]. In our group of CRs with VVP bypasses, 11% of patients developed temporary renal failure. No one required permanent dialysis.

Venous outflow problems have been debated as a potential downside of the PB technique, leading to venous congestion and the impaired function of the graft [16]. Levi et al. demonstrated in a large group of 2000 patients that a considerable learning curve exists when tackling this challenge [17].

The PB technique with only one venous anastomosis regularly displays a shortened WIT compared with a CR, which requires two venous anastomoses. Mehrabi et al. reported on 500 cases of LT done in PB technique with a WIT of 45 minutes [5]. Our median WIT was 43 minutes. However, initial nonfunction was detected in only 5 cases, with retransplantation required and conducted for all of these patients.

Sakai et al. published a study in 2010 comparing LT in a PB technique without a venovenous bypass to a PB technique with a venovenous bypass and to CR with a venovenous bypass. The authors detected a highly significant advantage concerning the short- and long-term survival when avoiding the bypass. The bypass-associated morbidity and mortality were significant in their study, with incidents of fatal lung embolisms and cardiac failure during reperfusion leading to a 30-day mortality of more than 5%. The one-year survival was excellent for 93% of the patients with a PB technique without a bypass but significantly worse, at less than 80%, for the PB technique with a bypass and a CR with a bypass. It remains somewhat unclear how the use of an extracorporeal bypass system affects long-term survival to this extent [4]. The selection of the patients, with the healthier patients undergoing the PB LT without bypass, may have produced a relevant bias in this group. In our population, the 30-day mortality was only 3.4%, and the 1-year survival was 87.6%.

As organ shortage has prompted us to increasingly use grafts fulfilling extended donor criteria that are allocated via the rescue-allocation procedure, we believe that our excellent survival data despite this decline in organ quality may partly be attributed to the use of the VVP bypass. By generating stable and very controllable circumstances during the operative procedure, especially at the time of reperfusion, a considerable impact may be achieved regarding both patient and graft protection. Particularly for patients with high MELD scores, the potential cardiac strain may be alleviated by the employment of the bypass.

The additional costs generated by the use of an extracorporeal bypass system present an obvious disadvantage: In addition to the one-time investment of the (relatively simple) bypass pump device, each procedure generated costs of approximately $500 in our institution (tubes, valves, connection devices, and fluid). The bypass usually required a period of 2–2.5 hours, including the set-up and disassembly, and for this period of time the respective personal costs need to be added.

## 5. Conclusion

In light of the increasing organ shortage and the necessity of using ever more grafts fulfilling extended donor criteria to satisfy patient needs, a reappraisal of the employment of a venovenous and portal venous bypass may be considered for LT. Our results demonstrate that the currently postulated drawbacks of the bypass procedure may be outweighed by the positive effects described, especially the relief of portomesenteric congestion. Larger prospective and multicentred studies are recommended to clarify the issue.

## Figures and Tables

**Table 1:** Recipient characteristics.

Recipient	
Age	54 (19–72)
Gender	
Male	106 (65%)
Female	57 (35%)
LabMELD	17 (6–40)
LabMELD > 30	39 (24%)
Indication for LT	
Acute liver failure	20 (12%)
Alcoholic cirrhosis	45 (27%)
HCC	40 (24%)
PSC	13 (8%)
Graft failure	8 (5%)
HBV/HCV cirrhosis	13 (8%)
Others	24 (14%)

**Table 2:** Donor characteristics.

Donor	
Gender	
Male	83 (51%)
Female	80 (49%)
Age	58 (12–86)
BMI	28 (14–57)
CIT (min)	459 (100–883)
EDC	
*n* = 0	27 (55.1%)
*n* = 1	16 (32.7%)
*n* = 2	5 (10.2%)
*n* = 3	1 (2%)
Age > 65	46 (28%)
BMI > 30	47 (28%)
ICU > 7 days	29 (18%)
Sodium > 165 mmol/L	5 (3%)
Transaminase > 150 U/L	23 (14%)
Bilirubin > 3 mg/dL	7 (4%)

**Table 3:** Intraoperative data.

Transplantation	
Operation time (min)	269 (171–594)
WIT (min)	43 (20–78)
Rescue allocation	77 (47%)
RBC	7 (0–56)
FFP	14 (1–75)

**Table 4:** Postoperative hospital stay and morbidity.

Postoperative data	
Groin seroma	2 (1%)
Renal failure	18 (11%)
Biliary leakage	5 (3%)
Thrombosis HA/PV	3 (2%)
Bleeding	28 (16%)
Primary nonfunction	5 (3%)
Re-LT	10 (6%)
ICU stay (days)	5 (1–196)
Hospital stay (days)	32 (14–299)

**Table 5:** Summary of current literature with comparison to our data.

Author	Technique	Renal failure	WIT (min)	RBC (*n*)
**Uniklinik RWTH Aachen**	**CR-B**, *n* =** 163**	**11%**	**43**	**7**

Schmitz et al., 2014 [3]	CR-B, *n* = 112		51	10
	CR, *n* = 126		45	8
	PB, *n* = 176		40	6

Sakai et al., 2010 [4]	CR-B, *n* = 104	35%	43	9
	PB-B, *n* = 148	25%	30	9
	PB, *n* = 174	15%	35	7

Mehrabi et al., 2009 [5]	PB, *n* = 500	6%	45	3

Khan et al., 2006 [6]	CR-B, *n* = 138	31%	44	5
	PB, *n* = 246	25%	43	4

Nishida et al., 2006 [7]	CR, *n* = 149, 80%B		45	18
	PB, *n* = 918, 20%B		35	13

Miyamoto et al., 2004 [8]	CR, *n* = 96		54	10
	PB, *n* = 71		63	4

Cabezuelo et al., 2003 [9]	CR-B, *n* = 20	50%		
	CR, *n* = 84	39%		
	PB, *n* = 80	18%		

Hesse et al., 2000 [10]	CR, *n* = 75, 67%B	24%		20
	PB, *n* = 72, 8%B	17%		11

Jovine et al., 1997 [11]	CR-B, *n* = 19	31%	60	
	PB, *n* = 20	0%	48	

Cherqui et al., 1994 [12]	PB, *n* = 61	13%		10
